# When it’s not safe to be me: employee authenticity mediates the effect of perceived manager psychopathy on employee well-being

**DOI:** 10.1186/s40359-023-01333-w

**Published:** 2023-10-09

**Authors:** Anna Sutton, Madeleine Stapleton

**Affiliations:** School of Psychology, University of Waikato, Private Bag 3105, 3240 Hamilton, New Zealand

**Keywords:** Psychopathy, Triarchic, Authenticity, Engagement, Burnout

## Abstract

**Background:**

Psychopathy in managers is often measured on global scales and associated with detrimental outcomes for subordinates, such as bullying and reduced well-being. Yet some features of psychopathy, like boldness, appear to have beneficial outcomes. Using the triarchic model of psychopathy, we differentiate between adaptive and maladaptive traits in managers and model their effects on employee engagement and burnout. In addition, we test the extent to which authenticity, known to ameliorate the effect of some negative experiences on well-being, might mediate the influence of managers’ perceived psychopathic traits on employee well-being.

**Methods:**

In a two-wave study, full-time employees (N = 246) reported on their manager’s psychopathic traits (boldness, meanness, disinhibition), their own authenticity and, six weeks later, their engagement and burnout.

**Results:**

In support of our hypotheses, manager boldness enhanced engagement and reduced burnout while meanness and disinhibition reduced engagement and increased burnout. Additionally, employee authenticity was a partial mediator of the effect of managerial psychopathy on engagement and burnout.

**Conclusions:**

Perceived psychopathic traits in managers have the potential to influence whether employees feel able to be their authentic selves at work, which consequently affects their well-being. A work culture that values authenticity can directly improve well-being and help employees to deal with managerial behaviour that stems from maladaptive psychopathic traits. We also highlight the importance of discriminating between constituent psychopathic traits to identify the potentially adaptive nature of the boldness element of psychopathy.

## Background

Psychopathy is associated with the need for power, and individuals higher on psychopathy may be drawn to positions that provide them with the opportunity to control or dominate others [1], such as managerial or leadership roles within work organisations. Indeed, a recent meta-analysis found that people with greater psychopathic tendencies are somewhat more likely to emerge as leaders but also to be less effective [2]. To illustrate, psychopathic personality is associated with bullying, conflict and reduced subordinate well-being in the workplace [3]. While the authors suggest that concern over psychopathic tendencies in managers and leaders in the popular press and research literature may be exaggerated, identifying the negative effects of psychopathy at work, and how those effects might be mitigated, is clearly of importance in building supportive workplaces which are higher performing.

Psychopathic personality is not, however, solely associated with detrimental outcomes in the workplace. Some of its constituent traits may be beneficial for employees [4]. There is extensive evidence to suggest that the effects of psychopathy are complex and sometimes contradictory, and models that distinguish between psychopathy’s central traits are needed [5]. In addition, recent work on the prevalence of psychopathy in the work environment has highlighted the limitations of using a taxonomic rather than dimensional approach [6]. Using the triarchic model of psychopathy, therefore, we aim to differentiate the effects of three constituent traits of psychopathy (boldness, meanness, disinhibition) on employee outcomes, specifically authenticity, engagement, and burnout.

Authenticity is the sense of ‘being true to oneself’ and is generally conceptualised in terms of accurate self-awareness combined with genuine self-expression [7, 8]. It is widely valued and recognised as contributing to well-being [9] and in the last decade there has been increased interest in the role of authenticity at work. Much of this interest has focused on authenticity in leaders and less attention has been paid to authenticity in employees occupying non-leadership roles [10]. Nonetheless, there is an emerging body of research highlighting the role that authenticity plays in enhancing employees’ well-being [11]. Despite the known benefits of authenticity, people sometimes selectively conceal aspects of their true selves at work, for example by strategically choosing to be inauthentic to meet job requirements or avoid interpersonal conflict [12] or because they feel controlled or judged by others [13]. Behaving authentically may therefore be a particular struggle for people who are vulnerable to social control, including that imposed on employees by managers. Manager psychopathic personality, with its association with abusive supervision, bullying, and conflict may therefore be a strong impetus for employee inauthenticity at work.

Yet for those employees who can be authentic, there may be significant benefits. Individual authenticity mediates the relationship between job demands and resources and well-being [14] and protects against the negative effects of interpersonal conflict [15]. In this paper, therefore, we propose that authenticity provides a partial mechanism for understanding the effects of perceived manager psychopathic personality on employee engagement and burnout.

### Psychopathy

As a clinical concept, psychopathy is defined as the combination of deviant behaviour and emotional or interpersonal detachment [16]. Although often referred to in terms of a clinical or forensic ‘type’, psychopathy is best conceptualised in terms of continuous traits, which are present at sub-clinical levels in the normal population [17]. That is, the traits that make up psychopathic personality can be measured in the non-clinical or non-forensic population and it is only in extremes or in combination that they meet the clinical definitions of psychopathy [5]. Because psychopathic traits appear to be positively related to leadership emergence [2], psychopathy, along with Machiavellianism and narcissism (the so-called Dark Triad) has been the subject of increasing research attention in recent years. Of the Dark Triad, psychopathy has the potential to be the most destructive, yet is the least explored [3].

Psychopathy is of particular interest in leadership studies and the workplace because of the extensive impact that more psychopathic leaders may have on their subordinates and organisations [17]. The quality of the relationship between leaders and their subordinates is known to be critical to follower success and satisfaction and those who score higher in psychopathy typically have poorer interpersonal relationships [18]. Employees with more psychopathic supervisors report greater levels of psychological distress, emotional exhaustion, and burnout [3, 19, 20].

Yet the effects of psychopathic personality on various work outcomes are often inconsistent or of small overall size [21], and this may be due to psychopathy typically being measured globally, using overall scores rather than constituent traits. A recent study tested several potential models of ‘successful’ psychopathy, which reflect the idea that psychopathy can be associated with beneficial outcomes [5]. Of the tested models, the differential configuration model, in which the varying effects of psychopathy are due to different configurations of its central traits, emerged as the most well supported. The triarchic model is one such model, developed as an integrating framework for disparate conceptualisations in the psychopathy literature [16]. The triarchic model describes psychopathic personality in terms of three main phenotypic dispositions: boldness, meanness, and disinhibition [16, 22]. Boldness is conceptualised as the tendency towards being emotionally resilient and socially dominant, demonstrating confidence and being more likely to take on risks. Meanness captures characteristics around aggressive resource-seeking, such as contempt and lack of empathy for others, exploiting people and using cruelty and destructiveness to build one’s own power base. Finally, disinhibition represents problems with impulse control, including a lack of restraint and emotional regulation as well as increased hostility towards and mistrust of others [16, 23].

The triarchic model is particularly useful in non-clinical settings and research utilising other-report because it conceptualises psychopathy in terms of observable characteristics that knowledgeable others might be expected to be able to report on. This more nuanced understanding of psychopathy, allows researchers to consider both potentially adaptive and maladaptive effects of psychopathy in various contexts [24]. The triarchic model has also recently proved valuable in distinguishing subtypes of psychopathic personality, including ‘successful’ psychopathy [25].

Despite the well evidenced negative effects of psychopathy in the workplace, senior managers have reported significant levels of psychopathy [26]. Their ability to attain such high-level roles suggests that psychopathic traits may be adaptive in some way in the workplace, just as research indicates that psychopathic traits may provide an evolutionary advantage. For example, psychopathy is advantageous in hostile psycho-social environments and associated with ‘faster’ life strategies that prioritise immediate risky gains over longer-term advantages [27]. Selfish risk-taking, an element of psychopathy, can also be associated with success in situations where survival is threatened [28]. In a fast-paced business world focused on immediate profits rather than long-term sustainability and where people’s livelihoods may be threatened, it is easy to see how psychopathic tendencies could be beneficial.

There is, of course, significant complexity in evaluating the ‘adaptiveness’ of psychopathy at work. While meanness and disinhibition reflect interpersonal antagonism and impulsivity, and are therefore considered maladaptive in terms of their outcomes and effect on others, the boldness dimension includes potentially positive adjustment features. It is therefore the most controversial of the triarchic traits, with some scholars disputing its relevance to psychopathy [29]. In addition, psychopathic traits may be directly associated with positive managerial competencies (for example, risk taking and innovation). Some psychopathic characteristics, such as lack of empathy, may be interpreted as positive, business-relevant traits such as the ability to make ‘tough’ unpopular decisions [30].

While overall psychopathy scores can predict certain successes in leadership behaviour and performance, boldness seems to be particularly important in distinguishing between adaptive and maladaptive consequences of psychopathy at work [31]. Boldness is known to be positively associated with using adaptive leadership styles as well as increased teamwork and employee engagement [32, 33]. Boldness in employees has also been shown to increase organisation citizenship behaviours [34]. In contrast, both meanness and disinhibition, representing the more maladaptive traits, are associated with unethical decision-making, counter-productive workplace behaviours and increased employee burnout [32–34].

### Engagement and burnout

Employee well-being is often operationalised in terms of engagement and burnout [35]. An engaged employee feels positive about their work and finds it fulfilling, a state of mind characterised by a sense of vigour, dedication to the role and absorption in the work [36]. Not simply the opposite of engagement, burnout is a distinct concept characterised by a sense of exhaustion, cynicism about work and a feeling of inefficacy in one’s job role [37]. There have been recent calls to investigate employee perceptions of their manager’s psychopathic personality [38] as previous work has demonstrated that manager psychopathy increases employee burnout [20], and particularly emotional exhaustion [19].

But when conceptualised and measured in terms of constituent traits, perceived manager psychopathy has variable effects. Boldness improves engagement, well-being, and job performance, while meanness and disinhibition act to increase burnout and reduce well-being and performance [33]. Evidence is starting to establish a link between psychopathic personality traits in managers and employee engagement / burnout, but the mechanism of this effect is still unknown. We propose that employee authenticity is a possible mechanism to explain how manager psychopathic personality traits translate into employee engagement and burnout.

### Authenticity as a potential mechanism

Meta-analysis has demonstrated a medium-strength relationship between authenticity and well-being across a range of studies and countries [9]. Authenticity is known to be a desirable state for employees, positively related to engagement and negatively related to burnout [11]. Being more authentic is associated with other favourable work outcomes too, including reduced strain [39], better job performance and satisfaction [40], and lower turnover [41]. It is not just authenticity in expressing individual aspects of the self (such as traits or values) that contributes to better work and life outcomes. Recent work has shown that authentic enactment of work roles and expression of collective identity further contributes to job satisfaction and well-being [42]. Authenticity also provides a buffering effect for the impact of negative experiences such as interpersonal conflict [15] and mediates the effects of job demands / resources on work outcomes such as engagement and burnout [14].

The Job Demands-Resources (JD-R) model provides a well-established framework for understanding how personal, social or systematic demands and resources at work can influence employee well-being via authenticity. Demands are aspects of the job that require effort and are associated with costs such as poorer well-being or burnout, while resources are aspects of the job that contribute to achievement of work goals and are associated with better outcomes such as engagement and higher well-being [43, 44]. Metin et al. [14] argue that authenticity acts as a mediator for job demands and resources by drawing on self-determination theory (SDT). SDT holds that people are authentic when their actions are autonomous and self-determined [45]. Job resources promote autonomy and self-determination and thereby a greater sense of authenticity and more positive outcomes. Authenticity thus acts as a mediator of the relationship between job resources and engagement or well-being [14]. Correspondingly, job demands limit autonomy and self-determination, thereby reducing authenticity and resulting in negative job outcomes [14].

Managers’ psychopathic personality can be a source of these demands and resources [33]. For example, boldness in a leader is posited to enable greater access to resources for subordinates as well as providing a structure that presents difficulties at work as challenges that can be overcome [33], leading to positive engagement or well-being. Similarly, boldness is associated with adaptive leadership styles [32], including those that are oriented to the needs of employees [33], and thereby provide employees with greater social and systematic resources.

In contrast, meanness and disinhibition are associated with negative or maladaptive leadership styles [32], acting as job demands. Meanness or disinhibition in a manager also leads to various forms of interpersonal mistreatment, which employees also experience as a form of job demand [46] and results in increased in burnout. Although authenticity is highly valued by both individuals and organisations, there are challenges in the workplace that may restrict employees’ ability to be authentic. First, psychopathic traits are associated with the motivation to dominate and control others [23] and people are more prone to hide their true selves in contexts that are perceived as controlling or unaccepting [13]. Similarly, controlling others involves reducing their social power and individuals who experience less social power are less able to be authentic [47]. Managers with psychopathic traits may therefore constrain employees’ ability to be authentic at work.

Second, employees may engage in strategic inauthenticity: choosing not to be true to themselves to achieve an important goal such as to avoid conflict with a supervisor [12] or improve their career prospects [48]. More psychopathic individuals may instigate conflict or take retribution on people, so psychopathic managers might be expected to create an environment in which employees are strategically inauthentic. Third, the importance of a sense of safety to both authenticity and engagement is well established [12, 49]. Given that psychopathy is associated with abusive supervision, it is likely that a psychopathic manager will reduce employees’ sense of safety, and thereby reduce authenticity and engagement and increase burnout.

For these reasons, meanness and disinhibition traits in managers can be conceptualised as demands: aspects of the job that require effort to deal with on the part of the employee. We propose that not only will the traits of meanness and disinhibition directly result in lower well-being-related outcomes, but this effect is also mediated through employees’ reduced authenticity.

### The present study

In summary, although there is evidence to suggest that global psychopathic personality in managers is negatively linked to employee well-being, there remains a need for research to identify the differential effects of psychopathic traits from subordinates’ perspectives [5, 38]. Using the triarchic model of psychopathy, we aim to evaluate these traits on a continuum in the normal working population and to distinguish between their differential effects in managers, on employees [5, 23].

In line with findings so far on the (mal)adaptive effects of the triarchic traits, we hypothesise distinct effects for boldness compared to meanness and disinhibition. Perceived boldness in managers, as the only psychopathic trait with the potential to be adaptive [50], is expected to have positive effects on employee outcomes (improving authenticity and thereby increasing engagement and decreasing burnout). In contrast, perceived meanness and disinhibition in managers are expected to represent the negative effects of psychopathy on employee outcomes (reducing authenticity and thereby decreasing engagement and increasing burnout). For each of these relationships, authenticity is expected to act as a mediator.

## Method

### Participants and procedure

New Zealand employees working across a range of industries were recruited through a research panel company as part of a larger data collection effort extending over three timepoints. Participants were required to work full-time and report to the same manager throughout the duration of the study. Those who met these criteria were sent an email with the URL link to the online information sheet. Once they gave consent to engage in the study, participants went on to complete the questionnaires. The full survey had a completion time of approximately 10–15 min, and was conducted over three timepoints: we report here on data from the first two collection points. At the first timepoint (T1), participants completed questionnaires assessing their perceptions of psychopathic traits (boldness, meanness, and disinhibition) in their direct manager, and their own authenticity. Six weeks later, at the second timepoint (T2), they completed questionnaires examining their own well-being at work, conceptualised as engagement and burnout.

*A priori* sample size was determined based on the requirements for the final timepoint study (not reported here) using a medium effect size (*f*^2^ = 0.15, based on previous findings in this area [33]), a desired power of 0.80 and an α level of 0.05 [51]. This gave a required sample size of 114 at the final timepoint of the data collection effort. With an estimated dropout rate of 40–45% (based on advice from the research panel company), this meant a required T1 sample size of 650.

The final number of participants who completed both T1 and T2 questionnaires was 246. Participant age ranged from 19 to 67 (M = 42.48, SD = 10.98); 55% of participants identified as female and 45% as male. Mean job tenure was 6.94 years (SD = 7.17) and participants had been reporting to their current manager for a mean of 3.99 years (SD = 4.25). Participants worked a mean of 39.60 h per week (SD = 4.03) and spent a mean of 25% of their week (SD = 37%) working remotely. The largest industry sectors represented were Professional, Scientific, Technical, Administrative and Support Services (16%), Education and Training (14%), and Health Care and Social Assistance (14%). All other industry sectors accounted for less than 10% of the sample.

### Measures

Participants completed measures regarding their perceptions of psychopathic traits in their manager and their own authenticity at T1, followed by measures of their engagement and burnout at T2.

#### Authenticity

The Integrated-Authenticity Scale (IAS) measures authenticity on two subscales: self-awareness (4 items, e.g. *For better or worse, I know who I really am*) and self-expression (4 items, e.g. *I always stand up for what I believe in*) on a scale from 1 = never to 5 = almost always [39]. All items may also be combined to produce an overall authenticity score (α > 0.80 in Knoll et al.’s study). In the present study, combining all items resulted in acceptable internal reliability (α = 0.64). With the removal of item 8, reliability increased to 0.73. To ensure more robust analysis, item 8 was therefore excluded from further analyses.

#### Manager’s perceived psychopathy

The Triarchic Psychopathy Measure (Work) [TriPM(Work), 33] is a short adaptation of the Triarchic Psychopathy Measure [52] used to assess psychopathy in the work context, with equivalent self- and other-report forms. Patrick’s original TriPM consisted of 58 items to assess the three dimensions of psychopathy, reduced to 21 items in the TriPM(Work). The Work scales exhibited very high convergent validity with the original scales as well as good predictive validity for work-based outcomes [53]. Despite this, we should note that the TriPM(Work) may not be reflecting the full domain of the original measure.

The TriPM(Work) other-report version evaluates employees’ perceptions of their manager’s psychopathy by asking them to assess their manager’s boldness (e.g. *My manager is well-equipped to deal with stress*), meanness (e.g. *My manager enjoys pushing people around sometimes*) and disinhibition (e.g. *My manager has missed work without bothering to call in*) with 7 items each on a scale from 1 = false to 4 = true.

#### Engagement

The two core elements of work engagement, vigour and dedication, were measured with 3 items each (e.g. *At my work, I feel bursting with energy* and *I am enthusiastic about my job* respectively) from the short version of the Utrecht Work Engagement Scale [54]. Items were rated on a scale from 1 = never to 7 = always (every day) and combined into an overall measure of work engagement.

#### Burnout

The two core elements of burnout, emotional exhaustion and cynicism, were measured using the Maslach Burnout Inventory - General Survey [55], with permission of the copyright holder. Five items each for emotional exhaustion (e.g. *I feel emotionally drained from my work*) and cynicism (e.g. *I doubt the significance of my work*) were rated from 1 = never to 7 = always, and combined into an overall burnout score.

### Data cleaning and analysis

Of the 271 eligible participants who completed both T1 and T2 questionnaires, we excluded 23 low-quality responses (those with > 5% missing data and those who completed the survey 50% faster than the median time [56]) and two multivariate outliers (based on Mahalanobis Distance, using a conservative chi-square cut-off of p < .001 [57]), leaving a final sample size of 246. Post-hoc power analysis on our final sample, using joint test of significance, confirmed a power of almost 1 to detect medium indirect effect sizes (*B* = 0.3) at an α level of 0.05 [58].

Cronbach alphas were calculated for all variables and were all > 0.7 (Table 1) indicating good to excellent reliability [59]. Descriptive statistics and correlations were calculated to illustrate the pattern of relationships between all variables and determine whether further regression analysis was indicated. Finally, we conducted mediated regression analysis to test the direct relationships between the predictor variables (perceived managerial psychopathy traits) at T1 and employee outcome variables (engagement and burnout) at T2, as well as the indirect mediated relationship between perceived psychopathic traits and employee outcomes via employee authenticity. Separate mediation regression models for each of the perceived psychopathic traits were tested, using PROCESS model 4 for SPSS [60] and 5,000 bootstrap samples to estimate indirect effects. We interpret the indirect effect sizes using the traditional Cohen guidelines (i.e. small 0.1, medium 0.3, and large 0.5) as recommended by Shrout and Bolger [61] combined with Kenny’s [62] suggestion that, because an indirect effect is the product of two effects, the effect size interpretations should be squared (i.e. small 0.01, medium 0.09, large 0.25).

## Results

Correlations between variables (Table 1) were generally higher than the average of r = .2 reported in recent summaries of psychological research [e.g. 63]. Most absolute intercorrelations in this study range from r = .28 to 0.40 and therefore do not exceed the 75th percentile of correlations reported in a recent analysis of over 30,000 published correlations [64].

The correlation between burnout and engagement (r = − .65) is notably higher than this and reflects debate over the conceptual and empirical distinction of these two variables. A study addressing this issue, using the same core elements of burnout and engagement as we used here, suggested that while burnout and engagement probably do not reflect different underlying processes (health impairment vs. motivation), they are differentially associated with specific job demands and resources [65]. Similarly, as in previous research using the other-report form of the TriPM(Work) [33], the correlations between boldness, meanness, and disinhibition were also fairly strong. The triarchic model of psychopathy does recognise some overlap in the dimensions [31], though as with burnout and engagement, there are differential associations between the three dimensions and various work outcomes [53]. We addressed the potential issue of high correlations as follows.

First, as we sought to investigate the influence of individual perceived managerial psychopathic traits, conceptualised as potential demands or resources to followers, we maintained the distinction between variables here, though we take care in our analysis and interpretation [65]. Second, and in support of this approach, the correlations do not reach the cut-off of r = .85 suggested as an indication of poor discriminant validity [66, 67]. Third, we do not enter highly correlated variables into regression models together, thereby avoiding any potential issues with multicollinearity.

Cronbach alphas indicated good to excellent internal reliability [59] and skewness and kurtosis values were all within acceptable ranges (-1 to + 1).

**Table 1:** Correlations and Descriptive Statistics for all Variables

	1	2	3	4	5	6
1. Boldness						
2. Meanness	− 0.66^**^					
3. Disinhibition	− 0.77^**^	0.79^**^				
4. Authenticity	0.28^**^	− 0.32^**^	− 0.31^**^			
5. Engagement (T2)	0.28^**^	− 0.24^**^	− 0.25^**^	0.33^**^		
6. Burnout (T2)	− 0.37^**^	0.40^**^	0.35^**^	− 0.30^**^	− 0.65^**^	
*Mean*	1.63	4.27	3.94	2.41	3.12	1.79
*SD*	0.61	0.88	1.24	1.48	0.57	0.73
* α*	0.83	0.92	0.88	0.73	0.94	0.95
*Skewness*	− 0.60	0.94	1.00	0.15	− 0.32	0.41
*Kurtosis*	0.25	0.31	0.40	− 0.57	− 0.22	− 0.47

*Note.* T2 = variables measured at time 2. ** p < .01

**Fig. 1:**
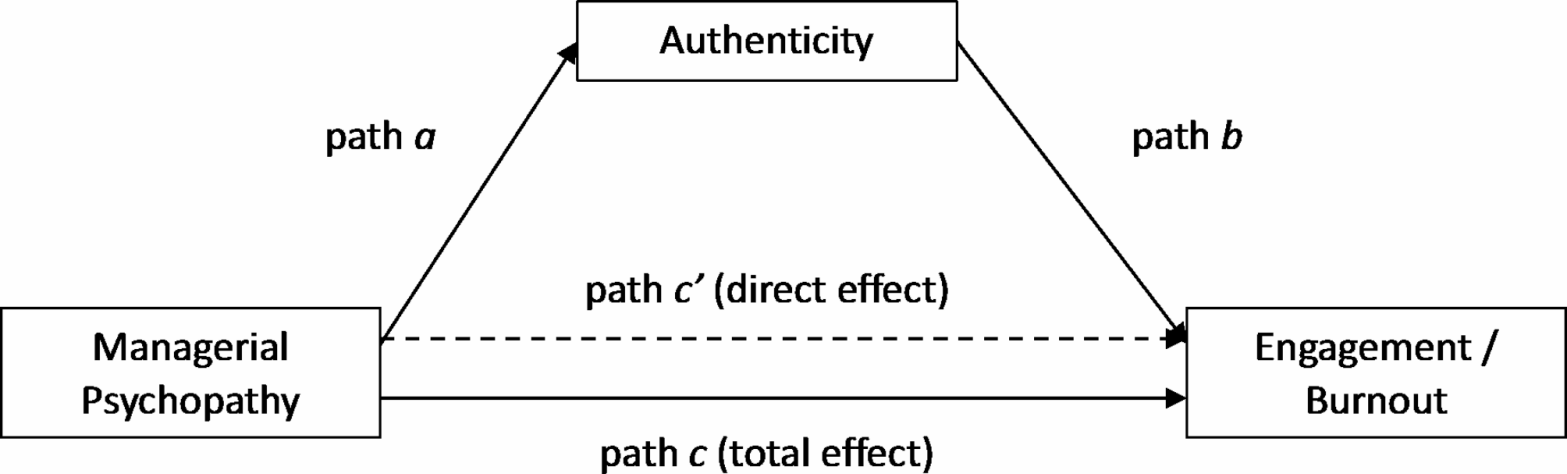
The mediation models tested in this study. *Note.* Path *a* indicates a direct effect of perceived psychopathic traits (boldness, meanness, or disinhibition) on employee authenticity. Path *b* indicates the direct effect of authenticity on engagement or burnout six weeks later. Path *c’* is the direct effect of perceived psychopathic traits on employee engagement / burnout and path *c* is the total effect

In total, six mediation models were tested: 3 perceived psychopathic traits (boldness / meanness / disinhibition) x 2 outcomes (engagement / burnout), with authenticity as the mediator (Fig. 1).

As hypothesised, managerial boldness and authenticity at T1 were both positively associated with engagement at T2, and the boldness-engagement relationship was partially mediated by authenticity (Table 2). Thus, employees with bolder managers were more authentic, which contributed to increased engagement six weeks later. The effects of manager meanness and disinhibition were also partially mediated by authenticity, though in this case, employees with meaner or more disinhibited managers were less authentic, with a concomitant reduction in engagement six weeks later. Indirect effects were statistically significant for all three models (as indicated by 95% CIs) and were of small-to-medium size [61, 62].

**Table 2:** Effects for authenticity mediating the link between perceived managerial psychopathy and employee engagement

	Boldness		Meanness		Disinhibition	
Path	B (SE)	95% CI	B (SE)	95% CI	B (SE)	95% CI
a	0.43*** (0.09)	[0.25, 0.62]	− 0.39*** (0.07)	[-0.53, − 0.24]	− 0.46*** (0.09)	[-0.63, − 0.28]
b	0.38*** (0.09)	[0.21, 0.56]	0.40*** (0.09)	[0.22, 0.57]	0.39*** (0.08)	[0.22, 0.57]
c	0.60*** (0.13)	[0.17, 0.70]	− 0.40** (0.11)	[-0.61, − 0.19]	− 0.51*** (0.13)	[-0.76, − 0.26]
c’	0.43** (0.13)	[0.34, 0.86]	− 0.25* (0.11)	[-0.46, − 0.04]	− 0.33* (0.13)	[-0.58, − 0.08]
Indirect effect	0.17* (0.06)	[0.07, 0.30]	− 0.15* (0.04)	[-0.25, − 0.08]	− 0.18* (0.05)	[-0.29, − 0.08]

*Note.* See Fig. 1 for mediation path details. SE = standard error (5,000 bootstraps for indirect effect). *p < .05. **p < .01. ***p < .001

Also in line with hypotheses, authenticity partially mediated the effects of perceived psychopathic traits on burnout (Table 3). Similarly to the positive findings for engagement, managerial boldness was associated with increased authenticity and a reduction in burnout six weeks later. Managerial meanness and disinhibition reduced employee authenticity, and subsequently increased burnout. Again, all indirect effects were of small-to-medium size.

**Table 3:** Effects for authenticity mediating the link between perceived managerial psychopathy and employee burnout

	Boldness		Meanness		Disinhibition	
Path	B (SE)	95% CI	B (SE)	95% CI	B (SE)	95% CI
a	0.43*** (0.09)	[0.25, 0.62]	− 0.39*** (0.07)	[-0.53, − 0.24]	− 0.46*** (0.09)	[-0.63, − 0.28]
b	− 0.35*** (0.10)	[0.25, 0.62]	− 0.32** (0.10)	[-0.52, − 0.12]	− 0.35*** (0.10)	[-0.56, − 0.15]
c	− 0.97*** (0.15)	[-1.27, − 0.66]	0.82** (0.12)	[0.59, 1.05]	0.86*** (0.15)	[0.57, 1.14]
c’	− 0.81*** (0.15)	[-1.12, − 0.51]	0.70*** (0.12)	[0.45, 0.94]	0.70*** (0.15)	[0.40, 0.99]
Indirect effect	− 0.15* (0.06)	[-0.28, − 0.05]	0.12* (0.04)	[0.05, 0.21]	0.16* (0.05)	[0.06, 0.28]

*Note.* See Fig. 1 for mediation path details. SE = standard error (5,000 bootstraps for indirect effect). *p < .05. **p < .01. ***p < .001

Of note, the direct effects of each psychopathic trait on burnout (*B* = − 0.81 for boldness and 0.70 for both meanness and disinhibition) appeared larger than the effects on engagement (*B* = 0.60 for boldness, − 0.40 for meanness and − 0.51 for disinhibition). To test this difference, we used Fisher’s Z test to compare correlations (which are equivalent to standardised betas in simple regression) from dependent samples [68]. The effect size of the relationship between boldness and burnout was significantly larger than between boldness and engagement (Z = -5.8, p < .001). Similarly, the relationship between meanness and burnout was larger than meanness and engagement ( (Z = 5.73, p < .001) and the relationship between disinhibition and burnout was larger than disinhibition and engagement (Z = 5.34, p < .001).

## Discussion

This short-term, two wave study investigated whether perceived manager psychopathy affects employee engagement and burnout, and the mediating role of authenticity in this relationship. Perceived psychopathic traits in managers had direct effects on employee engagement and burnout six weeks later. In addition, employee authenticity was found to partially mediate these relationships.

Notably, the three psychopathic traits differentially predicted employee outcomes. Manager boldness was associated with improved engagement and reduced burnout while manager meanness and disinhibition had the opposite effect, being associated with decreased engagement and increased burnout. Interestingly, the direct effects of the three perceived psychopathic traits were stronger for burnout than engagement. Previous work has also found a stronger relationship between psychopathy and indicators of diminished well-being (i.e., ill-being) compared with well-being [19, 33]. As suggested elsewhere [33], the Job-Demands Resources model [69] may provide theoretical insight into these findings: managerial psychopathy may be acting more strongly as a job demand than a potential job resource. Specifically, while boldness in managers may be somewhat helpful as a resource to employees, acting to increase engagement, it may be of more use in reducing the impact of other job demands that lead to burnout. For example, boldness is associated with positive leadership styles and effective communication, so may be considered as a source of social support. Meanness and disinhibition, on the other hand, are associated with abusive behaviours, bullying and conflict so may limit the social support that employees can access. Thus, while meanness and disinhibition in managers may act to reduce engagement, their direct effect on increasing burnout is greater.

These findings provide support for the utility of the triarchic model of psychopathy in non-clinical settings [31], and the need for psychopathy models that use differential configurations of central traits to explain the effects of psychopathy on well-being outcomes [5]. Boldness appears to underlie much of the content of ‘successful’ psychopathy whereas meanness and disinhibition appear to be responsible for the deleterious effects. Recent work has demonstrated a similar pattern of results for organisational citizenship and counterproductive workplace behaviours [34]. Similarly, high scores on meanness and disinhibition (but not boldness) were key to the secondary psychopathy subtype identified in Guo et al.’s [25] work: a subtype associated with higher anxiety and difficulty with emotion regulation. Boldness, in contrast, was more closely associated with the ‘successful’ psychopathy subtype. This study contributes to the building evidence that psychopathic personality has both adaptive and maladaptive effects at work and that closer examination of the constituent traits is needed to disentangle the often conflicting findings.

The results also provide further evidence that boldness should not be excluded when psychopathic personality is evaluated in the work context. Psychopathy is a complex construct that is associated with certain successes in life as well as distinct negative outcomes [50]. Using measures which include boldness, such as the TriPM(Work), ensures that we do not focus exclusively on the maladaptive traits associated with psychopathy and thereby miss what may help those with psychopathic traits succeed despite the potentially damaging consequences for others.

Recognising that psychopathy should not be assessed in organisations using global scales precludes screening potential managers for global psychopathy as it could unfairly exclude those with higher levels of boldness [22], seen here to be beneficial to subordinates. Because those with psychopathic traits are more likely to emerge as leaders [2], it is important for organisations to evaluate the specific constellation of psychopathic traits: boldness can be beneficial but meanness and disinhibition have both direct and indirect negative effects on employee well-being. We also recommend that organisations not rely exclusively on self-report of psychopathic traits but consider how employees’ perceive their managers, as we have demonstrated here a practically-relevant effect of those perceptions on employee reports of their burnout and engagement.

The use of the triarchic model also encourages the view of psychopathy as ‘treatable’ [50] as it emphasises observable behaviours rather than immutable internal attributes. Therefore, training aimed at so-called psychopathic managers is plausible. According to our findings, training that might help managers reduce mean and disinhibited behaviours could have substantial effects in increasing employee engagement and reducing employee burnout.

Our findings also confirm that individual authenticity is positively related to workplace well-being six weeks later, in line with previous longitudinal studies [70]. This means that employees who can express their true selves when working are not only more engaged in their work but also experience fewer symptoms of burnout. Further, in response to calls for longitudinal designs to improve our understanding of the dynamics of authenticity at work [14], we have demonstrated that authenticity partially mediates the relationship between perceived manager psychopathy and employee work-related well-being. Previous within-person research has shown that coping styles mediate the relationship of psychopathy with well-being [71] and our work adds to the understanding of the mechanisms of psychopathy by considering how internal resources such as authenticity may help mediate the effect of managerial psychopathic personality.

Wessell et al. [42] note that authenticity involves more than simply being aware of and expressing one’s personality or values: it also includes enacting a role in a way that feels true to oneself. The original definition of engagement at work included the sense of bringing one’s whole self into the work role, that is, being authentic [72], and the importance of a sense of safety to authenticity is well established [49]. Bolder managers seem to be helping to create a work environment in which employees can engage authentically with their work role. In contrast, meaner and more disinhibited managers are known to be more likely to use abusive supervision styles [33], reducing the sense of safety at work. This study indicates that these managers also reduce individual authenticity. Instead of feeling able to be true to themselves, employees in this situation are likely to behave more inauthentically, resulting in deleterious effects on engagement and burnout.

Authenticity is an important element of well-being and personal growth [73, 74] and can reduce the negative effects of experiences such as interpersonal conflict on well-being [15]. From the employee’s perspective, the more authentic they are, the more they can attenuate the negative effect of managers’ meanness and disinhibition on their well-being. The effect of individual dispositions on relationships between managerial behaviour and employee outcomes is an important growth area of organisational research. Employee dispositional forgiveness, for example, was recently shown to moderate the relationship between manager’s abusive supervision and employee attributions [75]. Similarly, we have demonstrated that employee dispositional authenticity is influential in how perceived managerial psychopathy influences engagement and burnout.

The gathering evidence indicates that authenticity may serve as a personal resource that employees can draw on to meet their work demands, which may include meaner or more disinhibited managers. Establishing a working culture that values and promotes authenticity could therefore not only directly improve well-being but also potentially help employees to deal with difficult individual managers who display tendencies towards meanness or disinhibition. We recognise that this may be difficult, as behaving authentically is less common in work than non-work contexts [76] and managers cannot simply induce authenticity in their employees. However, early indications suggest that it could be beneficial to incorporate interventions [e.g. mindfulness 77] to cultivate employee authenticity.

### Limitations and future research

In response to calls for further investigation of employee perceptions of psychopathy [38], we used an other-report measure of managerial psychopathic personality traits. This measure is likely to be subject to halo effects, whereby a leader who is seen as (in)effective is rated more positively or negatively overall [78]. Similarly, the pervasiveness of the fundamental attribution error suggests that an employee who is experiencing burnout may well attribute the cause to the manager’s behaviour rather than their own internal factors. While we controlled for this somewhat by having a time-lagged design that measured perceived psychopathic traits weeks before measuring burnout, the possibility of bias remains. Our findings therefore await replication using manager self-report or indeed, triangulated measures of psychopathy. We would expect similar results, as meta-analysis of self- and other-report personality measures has demonstrated good agreement between raters [79]. In addition, naïve participants are able to judge two of the three triarchic traits in peers after just a short interaction [80], and it is therefore likely that employees who have worked with their managers over the course of several years will be able to make even more accurate judgements.

Presentation of questionnaires was unfortunately not randomised within each timepoint for the data collection in this study, and thus we cannot rule out potential order effects. Future research evaluating similar mediation models would benefit from counterbalancing the survey completion order within each timepoint.

Several significant limitations exist in the use of mediation analysis, such as systematic bias in error estimation, underlying (unstated) assumptions that mediators are randomly dispersed in the population, and decisions that researchers make in terms of how to use or interpret the models [81, 82]. For this reason, we recommend that the findings here are further tested using experimental mediation designs rather than statistical models [81]. While experimental manipulation is challenging in the study of individual differences such as psychopathic personality traits, recent studies have demonstrated that some individual difference variables can be influenced [83] and certainly there is extensive evidence that one’s perceptions of another’s attributes or even one’s own personality can also be manipulated [84, 85].

The correlations between the TriPM(Work) scales are higher in this study than studies using the full self-report TriPM [23], though they are in line with previous work using the other-report version of the TriPM(Work) [33]. As noted above, this may be due to a halo effect. While the correlations in this study remain below the suggested cut-off of *r* = .80 that may indicate collinearity, we chose to remain cautious with the regression analyses and avoided including all three psychopathy traits in a single equation. Psychopathy is conceptualised as a constellation of traits, but the three traits are conceptually distinct, associated with different outcomes and even showing some opposing relations [16]. Therefore, as other researchers have done [e.g. 32], we considered the individual contributions of boldness, meanness, and disinhibition. Future research could consider combinations of these traits, and their influence on employee well-being via authenticity.

In addition, authenticity was measured solely in terms of individual self-awareness and self-expression, but recent work has demonstrated the incremental validity of using wider measures of authenticity, which include the authentic expression of our social roles and group memberships (Wessel et al., 2020). Thus, future research could explore whether these more collective elements of authenticity act as a buffer against meaner and more disinhibited individuals in a wider range of contexts, in much the same way that social support can help to reduce the effects of stress.

## Conclusion

In summary, perceived psychopathic traits in managers have the potential to influence whether employees feel able to be their authentic selves at work, which consequently affects their well-being. While previous research has shown links between manager psychopathy and employee well-being, this study is the first to identify employee authenticity as a partial mediator explaining the influence of managerial boldness, meanness, and disinhibition on employees’ engagement and burnout. Considering that managerial boldness evinced differential relations with employee authenticity, engagement, and burnout, when compared to managerial meanness and disinhibition, the study findings further emphasise the need for research to continue to parse psychopathy into its constituent traits when evaluating its implications in the work context. We encourage future research to continue to adopt this nuanced understanding of how psychopathy manifests in non-clinical settings, by considering psychopathy from the triarchic model perspective.

## Data Availability

The dataset analysed in this paper is available on the Open Science Framework database: https://osf.io/hdr9b/?view_only=369684f5b9864b738ce84e3ef9833005.
